# Impact of Cannabinoids on Symptoms of Refractory Gastroparesis: A Single-center Experience

**DOI:** 10.7759/cureus.6430

**Published:** 2019-12-20

**Authors:** Benjamin Barbash, Dhruv Mehta, Mohamed Tausif Siddiqui, Lavneet Chawla, Brad Dworkin

**Affiliations:** 1 Department of Gastroenterology, Bridgeport Hospital, Bridgeport, USA; 2 Department of Gastroenterology and Hepatology, Westchester Medical Center, Valhalla, USA; 3 Digestive Disease and Surgery Institute, Cleveland Clinic, Cleveland, USA; 4 Department of Hospital Medicine, Bridgeport Hospital, Bridgeport, USA; 5 Division of Gastroenterology and Hepatobiliary Diseases, Westchester Medical Center/New York Medical College, Valhalla, USA

**Keywords:** cannabis, gastroparesis cardinal symptom index, refractory gastroparesis, abdominal pain

## Abstract

Background and aims

Cannabinoids are increasingly used for medicinal purposes, including neuropathy. Gastroparesis is a neuromuscular disorder and neuropathy plays a large role in its pathogenesis. It is thus reasonable that cannabinoids can serve a beneficial role in the management of gastroparesis. Our study evaluates the effect of cannabinoids on gastroparesis symptoms.

Methods

Twenty-four (n=24) patients with gastroparesis and refractory symptoms were selected from a single gastroenterology practice associated with a tertiary care medical center. The ‘Gastroparesis Cardinal Symptom Index' (GCSI) and an analog scale rating abdominal pain were applied to prospectively assess the effect of cannabinoids, in the form of dronabinol and medical cannabis, on refractory gastroparesis symptoms. Patients completed a GCSI form and rated their abdominal pain, before and after treatment. There was a minimum of 60 days of cannabinoid use between reporting intervals. Total composite GCSI symptom scores, GCSI symptom subset scores, and abdominal pain scores were calculated before and after treatment.

Results

A significant improvement in the GCSI total symptom composite score was seen with either cannabinoid treatment (mean score difference of 12.8, 95% confidence interval 10.4-15.2; p-value < 0. 001). Patients prescribed marijuana experienced a statistically significant improvement in every GCSI symptom subgroup. Significant improvement in abdominal pain score was also seen with either cannabinoid treatment (mean score difference of 1.6; p-value <0.001).

Conclusions

Cannabinoids dramatically improve the symptoms of gastroparesis. Furthermore, an improvement in abdominal pain with cannabinoids represents a breakthrough for gastroparesis-associated abdominal pain treatment, for which there are currently no validated therapies.

## Introduction

Gastroparesis is a chronic neuromuscular disorder that results in delayed gastric emptying in the absence of mechanical obstruction [1]. The condition causes many difficult-to-treat symptoms, including nausea, vomiting, early satiety, bloating, anorexia, and abdominal pain [2]. Idiopathic gastroparesis and diabetes mellitus account for the majority of gastroparesis cases, though other etiologies are well-described, including post-surgical, collagen-vascular disease, neuromuscular disorders (e.g. Parkinson disease, multiple sclerosis), malignancy, hypothyroidism, drug-induced, and end-stage renal disease [3].

The socioeconomic impact and detrimental effect on the quality of life resulting from gastroparesis is significant and is increasing in recent years. Over 10% of patients reported being disabled due to their condition, while many other gastroparetics report missing significant work days and income [4-6]. Hospitalizations for gastroparesis increased by >150% over 1995-2004 and >300% over 1997-2013, with hospitalizations for gastroparesis resulting in extended lengths of stay as compared to hospitalizations for other upper gastrointestinal (GI) disorders [7-8]. Although the exact prevalence of gastroparesis in the United States is unknown, it seems to be an under-diagnosed and thus under-treated disorder [9].

The pathophysiology resulting in gastroparesis symptoms is not fully understood, although neuropathy likely plays a large role in its pathogenesis. The impairment of normal phasic motor activity in the distal stomach produces the clinical manifestations related to delayed gastric emptying. The frequency and direction of the phasic motor activity is regulated by the gastric slow wave, a rhythmic electrical oscillation, which is generated by the interstitial cells of Cajal in the proximal gastric body - this area thus is known as the "pacemaker" zone of the stomach [10]. Treatments for gastroparesis often focus on improving gastric motility, though data regarding the correlation between the degree of delayed gastric emptying and symptom manifestations is variable [11]. Other treatments in gastroparesis aim to control its associated symptoms and include antiemetics and neuromodulators. The latter group of medications has often been used to treat abdominal pain specifically, despite a lack of efficacy seen in clinical trials [12].

Cannabinoids, primarily delta-9-tetrahydrocannabinol (THC) and cannabidiol (CBD), are becoming increasingly studied and used for medicinal purposes. Dronabinol, a synthetic THC analog, is used for nausea, vomiting, and anorexia in human immunodeficiency virus (HIV) and cancer though it has been used for symptom management in other conditions [13-14]. Medical marijuana in New York is permitted to treat neuropathy with severe nausea or severe pain [15]. Given the treatment indications for these cannabinoids, it is reasonable that they can serve a beneficial role in the management of gastroparesis. Newer, and more effective, treatment options for gastroparesis are needed and cannabinoids are a promising option. The study aimed at evaluating the effects of cannabinoids on refractory gastroparesis symptoms.

## Materials and methods

The effects of cannabinoids on gastroparesis symptoms were prospectively assessed in 24 patients. Patients were selected based on refractory symptoms from a single gastroenterology practice associated with a tertiary care medical center. Patients included in the study first required a confirmed diagnosis of gastroparesis via a nuclear gastric emptying study that displayed delayed gastric emptying, and esophagogastroduodenoscopies (EGD) that ruled out mechanical obstruction. Only patients with symptoms that were refractory to standard therapies for gastroparesis were included (this included dietary modification, medications (prokinetics, antiemetics, and neuromodulators), endoscopic therapy (e.g. botulinum toxin injections), and some patients had implantable gastric stimulators and/or surgical pyloroplasty).

Patients were prescribed either dronabinol, medical cannabis, or both, for symptom management. Those who received both treatments had them prescribed sequentially (dronabinol then marijuana) if dronabinol did not adequately relieve symptoms. Marijuana was prescribed as needed at varying THC:CBD ratios (at the discretion of the cannabis dispensary) and taken via vaporized inhalation or sublingual drops. The dosage of dronabinol ranged from 2-10 mg twice daily to four times daily. Patients completed a GCSI form, a validated symptom index for gastroparesis, before and after treatment (Table 1).

**Table 1:** Gastroparesis Cardinal Symptom Index (GCSI)

Symptom Subscale	Symptom	None	Very Mild	Mild	Mod	Severe	Very Severe
Nausea/ Vomiting	Nausea	0	1	2	3	4	5
	Retching	0	1	2	3	4	5
	Vomiting	0	1	2	3	4	5
Fullness/ Early Satiety	Stomach fullness	0	1	2	3	4	5
	Not able to finish meals	0	1	2	3	4	5
	Fullness after eating	0	1	2	3	4	5
	Loss of appetite	0	1	2	3	4	5
Bloating/ Distention	Bloating	0	1	2	3	4	5
	Belly visibly larger	0	1	2	3	4	5

They also rated their abdominal pain before and after treatment using a 1-5 analog scale. There was a minimum of 60 days of cannabinoid use between reporting intervals. Total composite GCSI symptom scores, GCSI symptom subset scores, and abdominal pain scores were calculated before and after treatment.

Primary outcomes included changes in GCSI composite symptom scores, changes in GCSI individual symptom subset scores, as well as changes in abdominal pain scores for cannabinoid therapy. The secondary outcome was differences in GCSI composite scores, individual GCSI symptom subset scores, and abdominal pain scores between marijuana and dronabinol.

We performed bivariate analysis with Pearson’s chi-squared test to compare the demographic differences in study groups. To compare the differences in the composite scores (pre and post-treatment) for each group, we utilized a paired-sample t-test and reported 95% confidence intervals for mean score differences. We used an unpaired t-test to analyze the differences in treatment outcomes between the study groups. We reported two-sided p-values and a p-value less than 0.05 was considered statistically significant. All statistical analyses were performed using the SPSS Statistical software v25.0 (IBM Corp, Chicago, Illinois).

## Results

Baseline characteristics were collected for all 24 patients in the study (Table 2). The mean age of the study population was 44.8 years. Twenty (83.3%) females and four (16.7%) males were included in the study. The etiology of gastroparesis in the study population were as follows: idiopathic (11), diabetes (8), post-surgical (2), collagen vascular disease (2), and neuromuscular disease (1). Of the 24 patients, 1/3rd (eight patients) had gastric neurostimulator placed in the past. Six patients were prescribed dronabinol, 10 were prescribed marijuana, and eight were prescribed dronabinol followed by marijuana. All 24 patients completed both pre- and post-treatment GCSI questionnaires and abdominal pain scales.

**Table 2:** Baseline patient characteristics

Gender (n) (%)	
Male	4 (16.7%)
Female	20 (83.3%)
Mean Age (years)	44.87 (24-81)
Gastroparesis Etiology (n) (%)	
Idiopathic	11 (45.9%)
Diabetes	8 (33.3%)
Post-Surgical	2 (8.3%)
Neuromuscular Disease	1 (4.2%)
Collagen Vascular Disease	2 (8.3%)
Gastric Neurostimulator (Enterra ^©^) (n) (%)	8 (33.3%)
Cannabinoid Prescribed (n) (%)	
Dronabinol	6 (25%)
Marijuana	10 (41.7%)
Dronabinol then Marijuana	8 (33.3%)

**Figure 1:**
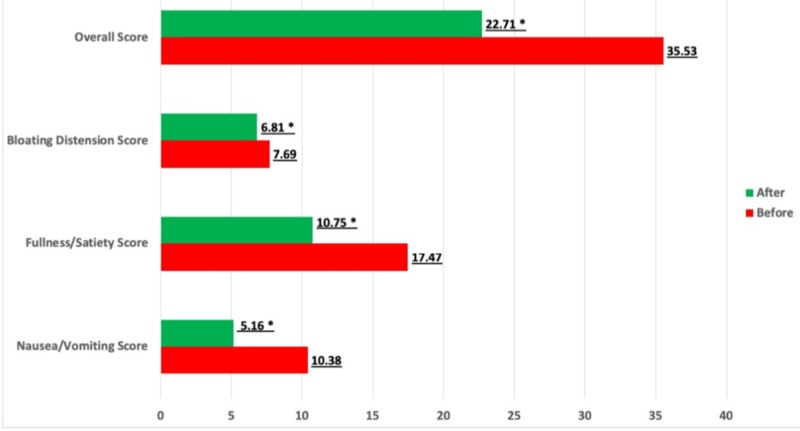
Comparison of Gastroparesis Cardinal Symptom Index (GCSI) composite symptom score and GCSI symptom subgroup scores before and after either cannabinoid treatment * indicates p-value < 0.05

Initial analysis was performed for the entire study population. Paired sample T-tests were performed and showed a statistically significant improvement in the GCSI total symptom composite score in patients who received either cannabinoid treatment (mean score difference of 12.8, 95% confidence interval 10.4-15.2; p-value < 0. 001) (Table 3, Figure 1). Significant mean score differences were noted in the nausea/vomiting score (mean difference of 5.22; p<0.05), fullness/satiety score (mean difference of 6.72; p<0.05) and the bloating/distention score (mean difference of 0.88; p<0.05) (Table 3).

**Table 3:** Paired sample T-tests and differences of the mean for Gastroparesis Cardinal Symptom Index (GCSI) composite symptom score and symptom subgroups before and after either cannabinoid treatment

	Mean Difference	Std. Deviation	Std. Error Mean	95% Confidence Interval of the Difference		P-value (2-tailed)
				Lower	Upper	
Nausea/Vomiting Score	5.22	3.76	0.66	3.86	6.57	<0.001
Fullness/Satiety Score	6.72	3.78	0.67	5.36	8.08	<0.001
Bloating/Distension Score	0.88	1.43	0.25	0.36	1.39	0.002
Total Composite Score	12.81	6.70	1.18	10.40	15.23	<0.001

Table 4 shows a subset analysis with the mean differences for each GCSI component for patients receiving only marijuana. Patients prescribed marijuana experienced a statistically significant improvement in every symptom subgroup. The biggest mean difference was noted in the fullness/satiety score followed by nausea/vomiting score, abdominal pain score, and bloating/distention score (mean difference of 7.52, 5.35, 2.17, and 1.23 respectively; p<0.05). Subset analysis for the Dronabinol group experienced a statistically significant improvement in all symptom subgroups except ‘bloating/distention’ (Tables 4-5).

**Table 4:** Paired sample T-tests and differences of the mean for changes in the Gastroparesis Cardinal Symptom Index (GCSI) symptom subset and abdominal pain scores before and after marijuana therapy

	Mean Difference	Std. Deviation	Std. Error Mean	95% Confidence Interval of the Difference		P-Value (2-tailed)
				Lower	Upper	
Nausea/Vomiting Score	5.353	3.278	0.795	3.668	7.038	<0.001
Fullness/Satiety Score	7.529	4.14	1.004	5.401	9.658	<0.001
Bloating/Distension Score	1.235	1.522	0.369	0.453	2.018	0.004
Abdominal Pain Score	2.176	1.286	0.312	1.515	2.838	<0.001
Total Composite Score	16.294	6.899	1.673	12.747	19.841	<0.001

**Table 5:** Paired sample T-tests and differences of the mean for changes in the Gastroparesis Cardinal Symptom Index (GCSI) symptom subset and abdominal pain scores before and after dronabinol therapy

	Mean Difference	Std. Deviation	Std. Error Mean	95% Confidence Interval of the Difference		P-value (2-tailed)
				Lower	Upper	
Nausea/Vomiting Score	4.5	3.898	1.042	2.25	6.75	0.001
Fullness/Satiety Score	5.5	3.107	0.83	3.706	7.294	<0.001
Bloating/Distension Score	0.5	1.286	0.344	-0.243	1.243	0.169
Abdominal Pain Score	0.929	1.141	0.305	0.27	1.587	0.009
Total Composite Score	11.429	6.653	1.778	7.587	15.27	<0.001

**Figure 2:**
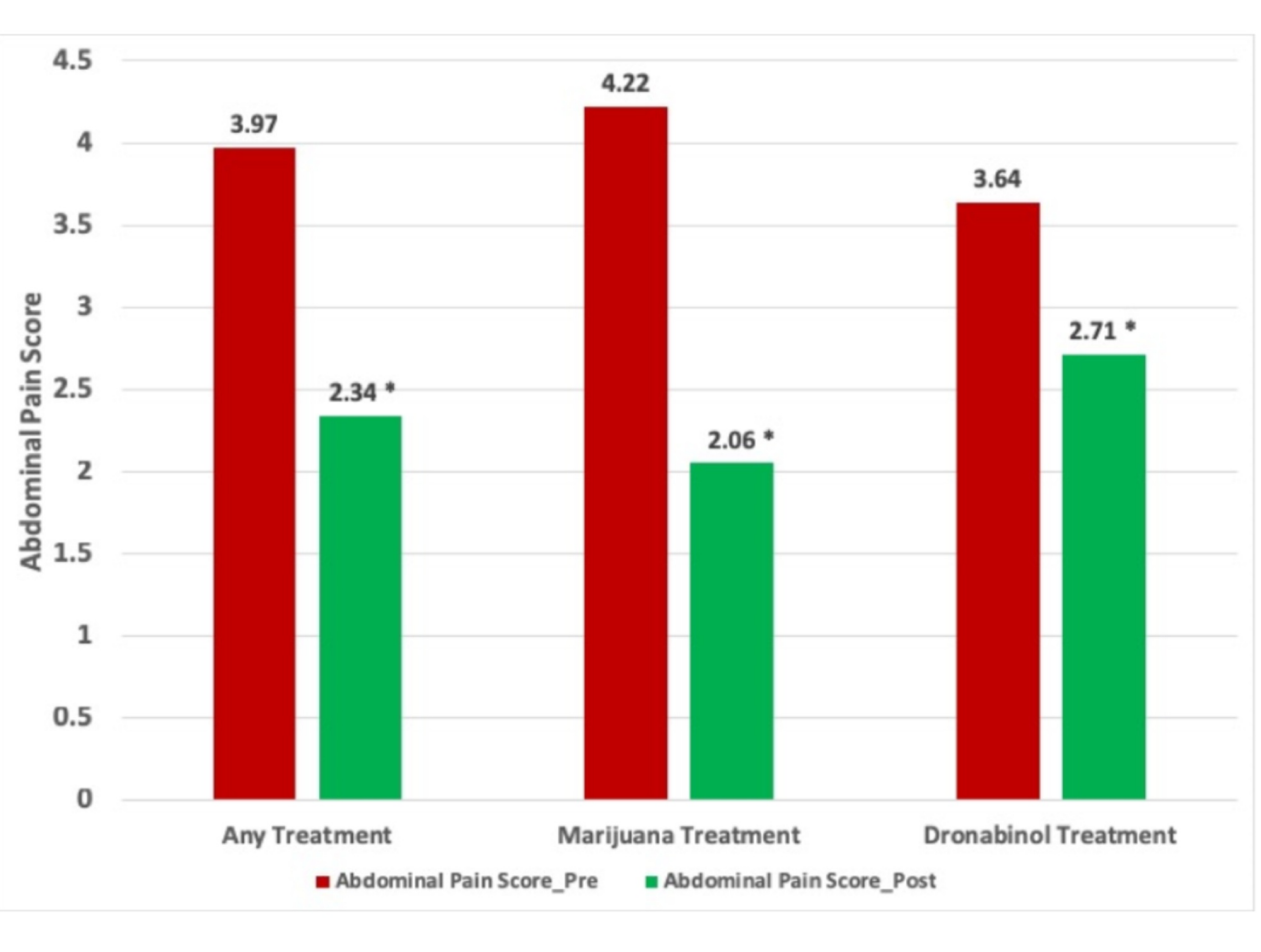
Comparison of abdominal pain scores before and after either cannabinoid treatment, marijuana treatment alone, and dronabinol treatment alone * indicates p-value <0.05

Paired sample T-tests were performed and a statistically significant improvement in the abdominal pain score was also seen in patients who received either cannabinoid treatment. When analyzed individually, both marijuana and dronabinol showed a statistically significant improvement in abdominal pain scores as well (p<0.05) (Figure 2).

**Figure 3:**
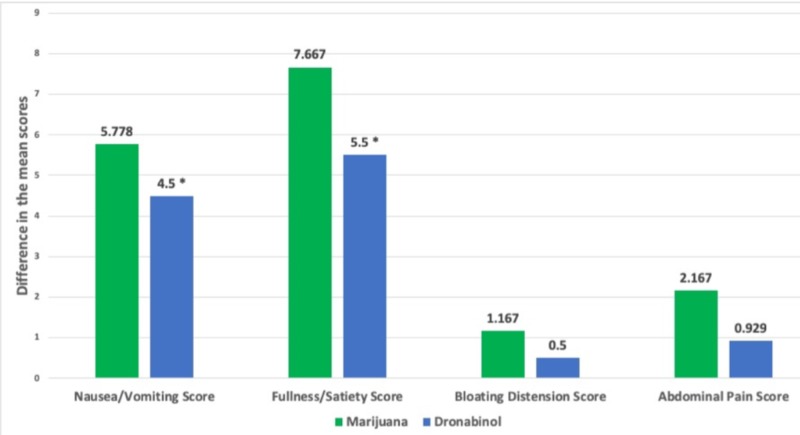
Comparison of the mean differences in symptom score improvement in each symptom category between marijuana and dronabinol * indicates p-value <0.05

To compare marijuana and dronabinol, pre- and post-treatment GCSI and abdominal pain scores were analyzed and unpaired sample T-tests were performed. Marijuana was superior to dronabinol in improving all symptoms, with statistical significance seen in the abdominal pain score and the total symptom composite score (includes the GCSI composite score and abdominal pain score) (p<0.05) (Figure 3).

## Discussion

There are only a small number of available treatments targeted for gastroparesis, and many of the treatment options are limited by side-effect profiles, restricted recommended treatment duration, and unsubstantiated efficacy data.

Prokinetics are often used because they target the underlying pathophysiology of the disease process, although symptom severity does not necessarily correlate with gastric emptying times [11,16]. Of the prokinetic medications, metoclopramide is the only Food and Drug Administration (FDA)-approved medication, for a duration of up to 12 weeks [2]. Erythromycin may be another effective promotility agent, though cardiac side effects and tachyphylaxis limit its use [17]. Domperidone is not FDA-approved in the USA. Many trials assessing the efficacy of promotility agents for gastroparesis have often had methodological flaws, though systematic review indicated that erythromycin and domperidone are most effective in improving symptoms [18].

Pain control is important in gastroparesis since up to 90% of gastroparetics report abdominal pain [19]. Neuromodulators such as tricyclic antidepressants are often used for pain symptoms, despite no benefit being observed in a major clinical trial assessing nortriptyline for gastroparesis symptoms [12].

Endoscopic and surgical interventions are not well-studied in gastroparesis and data regarding effectiveness are scarce and often conflicting [19]. Given the lack of data supporting the efficacy of these treatments, along with an inherent increased risk to the patient since these are procedural or surgical interventions, these are ‘last options’ in patients with severe and refractory symptoms.

Cannabinoids represent a new treatment in this difficult-to-treat, burdensome condition with minimal data-supported treatment options. By extrapolating from the indications for treatment with dronabinol and by utilizing the newly approved indications for medical cannabis in New York, cannabinoids clearly can benefit patients suffering from gastroparesis symptoms.

We demonstrated that cannabinoids dramatically, and significantly, improve all symptoms of gastroparesis. Furthermore, abdominal pain was significantly improved with cannabinoids. This role in pain management represents a breakthrough for gastroparesis-associated abdominal pain treatment, for which there are currently no validated therapies.

When both cannabinoid treatments are analyzed individually, both therapies resulted in an improvement in the GCSI composite symptom score, all GCSI symptom subset scores, and abdominal pain scores, though ‘bloating/distention’ with dronabinol treatment did not reach a statistical significance. When compared directly, marijuana was superior in improving overall symptoms, though this seems mainly driven by significant superiority in improving abdominal pain.

Our study represents one of the first studies analyzing cannabinoids for the treatment of refractory gastroparesis symptoms [20]. Therapies for gastroparesis have rarely shown such beneficial results, and cannabinoids could represent a major treatment breakthrough.

Limitations of this study include the small sample size due to the study design and the lack of a placebo-controlled or blinded methodology, though this would be difficult for marijuana studies. Also, the length of treatment prior to reassessing symptoms varied between patients, as it is unclear how long it would take to reach a therapeutic benefit from cannabinoid treatment. Furthermore, there was no standardized concentration of THC and CBD in the marijuana prescribed. At this point in New York, the THC:CBD ratios are variable. A trial-and-error process ensues, where the patient may adjust the THC:CBD ratio until maximal symptom relief and minimal side effects are achieved. It appears that THC was required to benefit the patients, but no definitive THC:CBD ratios can be recommended at this time.

## Conclusions

In conclusion, cannabinoids dramatically improve refractory gastroparesis symptoms, including abdominal pain. Marijuana may be superior to dronabinol in improving these symptoms, though both cannabinoids seem to be promising as novel therapeutic options in gastroparesis. Further studies should be conducted to confirm the efficacy of cannabinoids in refractory gastroparesis, and focus should be applied to optimal THC:CBD dosing, long-term efficacy, and sustainability of symptom improvement, as well as the side-effects of chronic cannabinoid use.
